# Supplementary material for: A Prognostic Merit of Statins in Patients with Chronic Hemodialysis after Percutaneous Coronary Intervention—A 10-Year Follow-Up Study
Source: J Clin Med. 2022 Jan 13;11(2):390. doi: 10.3390/jcm11020390 (PMC8780570; doi:10.3390/jcm11020390)

Supplementary Table S1: Covariates included in the models of multivariate Cox proportional hazard analyses

	**Model 1**	**Model 2**	**Model 3**
Age, years old	x	x	x
Gender, male	x	x	x
BMI[1]	x	x	x
Diabetic nephropathy	x	x	x
Beta blockers		x	x
LDL-C[2], mg/dL		x	x
hs-CRP[3], mg/L			x

Abbreviations: 1: BMI, body mass index; 2: LDL-C, low density lipoprotein-cholesterol; 3: hs-CRP, high-sensitivity C-reactive protein.

Supplementary Table S2: Interactions among statin therapy and LDL-C

Model 2*

p-value of interaction between	for CV death	for All-cause death	for 3P-MACE
Statin and LDL-C	0.19	0.14	0.54

Model 3**

p-value of interaction between	for CV death	for All-cause death	for 3P-MACE
Statin and LDL-C	0.17	0.01	0.51

*Model 2 included age, gender, BMI, diabetic nephropathy, beta blockers and LDL-C. **Model 3 further included serum hs-CRP level in addition to the variates of Model 2.

Supplementary Table S3: Univariate-unadjusted Cox proportional hazard analysis for predicting cardiovascular mortality, all-cause mortality and 3P-MACE

Cardiovascular mortality

	HR[1]	95%CI[2]	P-value
Age	1.03	1.00 - 1.06	**0.033**
BMI[3] (1SD[4] elevation)	0.71	0.51 - 0.96	**0.025**
Male gender	1.62	0.65 - 5.39	0.33
Hypertension	1.85	0.75 - 6.17	0.20
Dyslipidemia	0.58	0.31 - 1.12	0.10
Diabetic nephropathy	1.71	0.94 - 3.23	0.077
Duration of hemodialysis (1 year longer)	1.01	0.97 - 1.04	0.59
History of smoking	1.15	0.65 - 2.09	0.64
Family history	0.88	0.43 - 1.68	0.71
Atrial fibrillation	1.38	0.53 - 3.02	0.48
Prior PCI[5]	1.15	0.56 - 2.20	0.69
Prior myocardial infarction	1.22	0.62 - 2.25	0.55
Prior CABG[6]	1.65	0.80 - 3.14	0.17
Peripheral arterial disease	1.50	0.74 - 2.84	0.24
Prior cerebrovascular disease	1.09	0.47 - 2.22	0.82
ACS[7]	1.55	0.75 - 2.96	0.22
Number of vessels (1 more vessel disease)	1.16	0.80 - 1.68	0.43
Beta blocker	0.55	0.30 - 0.99	**0.044**
CCB[8]	1.48	0.83 - 2.72	0.19
ACEI/ARB[9]	0.61	0.34 - 1.07	0.086
LDL-C[10], mg/dL (1SD elevation)	1.24	0.96 - 1.58	0.10
HDL-C[11], mg/dL (1SD elevation)	1.24	0.93 - 1.63	0.14
TG[12], mg/dL (1SD elevation)	0.51	0.32 - 0.76	**<0.001**
Non-FBG[13], mg/dL (1SD elevation)	1.18	0.90 - 1.47	0.22
Hemoglobin, g/dL (1SD elevation)	0.80	0.57 - 1.10	0.17
hs-CRP[14], mg/L (1SD elevation)	1.42	1.10 - 1.72	**0.011**
Albumin, mg/dL (1SD elevation)	1.14	0.83 - 1.57	0.41
eGFR[15], mL/min1.73 m^2 (1SD elevation)	1.19	0.88 - 1.56	0.24
Statin use	0.36	0.15 - 0.72	**0.003**

All-cause mortality

	HR[1]	95%CI[2]	P-value
Age	1.05	1.03 - 1.07	**<0.001**
BMI[3] (1SD[4] elevation)	0.71	0.56 - 0.89	**0.003**
Male gender	0.79	0.46 - 1.46	0.43
Hypertension	1.20	0.66 - 2.41	0.56
Dyslipidemia	0.72	0.45 - 1.20	0.20
Diabetic nephropathy	1.49	0.96 - 2.33	0.075
Duration of hemodialysis (1 year longer)	0.99	0.97 - 1.02	0.91
History of smoking	1.10	0.72 - 1.70	0.67
Family history	0.73	0.42 - 1.22	0.24
Atrial fibrillation	1.58	0.82 - 2.81	0.16
Prior PCI[5]	0.83	0.46 - 1.42	0.52
Prior myocardial infarction	1.21	0.73 - 1.92	0.45
Prior CABG[6]	1.48	0.86 - 2.43	0.15
Peripheral arterial disease	1.76	1.08 - 2.80	**0.024**
Prior cerebrovascular disease	1.43	0.83 - 2.36	0.19
ACS[7]	1.38	0.80 - 2.25	0.23
Number of vessels (1 more vessel disease)	1.21	0.92 - 1.60	0.16
Beta blocker	0.79	0.51 - 1.21	0.28
CCB[8]	1.12	0.73 - 1.72	0.61
ACEI/ARB[9]	0.75	0.49 - 1.15	0.19
LDL-C[10], mg/dL (1SD elevation)	1.10	0.90 - 1.33	0.35
HDL-C[11], mg/dL (1SD elevation)	1.22	0.98 - 1.49	0.072
TG[12], mg/dL (1SD elevation)	0.61	0.44 - 0.81	**<0.001**
Non-FBG[13], mg/dL (1SD elevation)	1.19	0.98 - 1.40	0.081
Hemoglobin, g/dL (1SD elevation)	0.84	0.66 - 1.06	0.13
hs-CRP[14], mg/L (1SD elevation)	1.52	1.27 - 1.76	**<0.001**
Albumin, mg/dL (1SD elevation)	0.86	0.68 - 1.09	0.21
eGFR[15], mL/min1.73 m^2 (1SD elevation)	1.28	1.04 - 1.55	**0.022**
Statin use	0.49	0.29 - 0.81	**0.004**

3P-MACE

	HR[1]	95%CI[2]	P-value
Age	1.02	0.99 - 1.05	0.15
BMI[3] (1SD[4] elevation)	0.87	0.66 - 1.13	0.30
Male gender	1.35	0.62 - 3.51	0.47
Hypertension	1.83	0.80 - 5.26	0.16
Dyslipidemia	0.68	0.39 - 1.25	0.21
Diabetic nephropathy	1.37	0.81 - 2.37	0.24
Duration of hemodialysis (1 year longer)	0.99	0.96 - 1.03	0.81
History of smoking	0.92	0.55 - 1.56	0.76
Family history	1.02	0.55 - 1.79	0.96
Atrial fibrillation	1.54	0.67 - 3.07	0.28
Prior PCI[5]	1.25	0.66 - 2.22	0.48
Prior myocardial infarction	1.07	0.58 - 1.89	0.81
Prior CABG[6]	1.47	0.76 - 2.66	0.24
Peripheral arterial disease	1.06	0.54 - 1.95	0.85
Prior cerebrovascular disease	0.81	0.36 - 1.61	0.57
ACS[7]	1.42	0.74 - 2.56	0.28
Number of vessels (1 more vessel disease)	1.10	0.79 - 1.53	0.58
Beta blocker	0.83	0.49 - 1.39	0.48
CCB[8]	1.09	0.64 - 1.84	0.76
ACEI/ARB[9]	0.55	0.33 - 0.93	**0.026**
LDL-C[10], mg/dL (1SD elevation)	1.18	0.93 - 1.47	0.18
HDL-C[11], mg/dL (1SD elevation)	1.21	0.93 - 1.55	0.14
TG[12], mg/dL (1SD elevation)	0.69	0.48 - 0.95	**0.021**
Non-FBG[13], mg/dL (1SD elevation)	1.17	0.92 - 1.42	0.19
Hemoglobin, g/dL (1SD elevation)	0.97	0.73 - 1.27	0.82
hs-CRP[14], mg/L (1SD elevation)	1.37	1.06 - 1.67	**0.019**
Albumin, mg/dL (1SD elevation)	1.16	0.87 - 1.54	0.32
eGFR[15], mL/min1.73 m^2 (1SD elevation)	1.16	0.88 - 1.49	0.28
Statin use	0.45	0.23 - 0.82	**0.008**

Abbreviations: 1: HR, hazard ratio; 2: CI, confidence interval; 3: BMI, body mass index; 4: SD, standard deviation; 5: PCI, percutaneous coronary intervention; 6: CABG, coronary artery bypass grafting; 7: ACS, acute coronary syndrome; 8: CCB, calcium channel blocker; 9: ACEI/ARB, angiotensin-converting enzyme inhibitor/angiotensin receptor blocker; 10: LDL-C, low density lipoprotein-cholesterol; 11: HDL-C, high density lipoprotein-cholesterol; 12: TG, triglycerides; 13: Non-FBG, non-fasting blood glucose; 14: hs-CRP, high-sensitivity C-reactive protein; 15: eGFR, estimated glomerular filtration rate.

Supplementary Figure S1: Consort diagram of the present study.

Patients who underwent their first PCI at Juntendo University Hospital between January 2000 and December 2016 were enrolled in the present study. Those who did not undergo maintenance hemodialysis at PCI were excluded.

Supplementary Figure S1

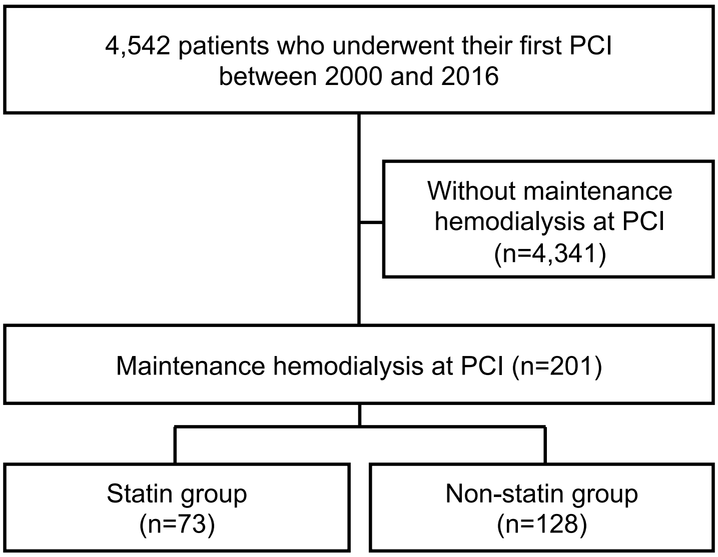

Supplement: Supplementary file 1 [file jcm-11-00390-s001.zip › jcm-1516436-supplementary.pdf]
